# Use of stepwise lung recruitment maneuver to predict fluid responsiveness under lung protective ventilation in the operating room

**DOI:** 10.1038/s41598-024-62355-x

**Published:** 2024-05-22

**Authors:** Eun Hee Chun, Mi Hwa Chung, Jung Eun Kim, Hye Sun Lee, Youngbum Jo, Joo Hyun Jun

**Affiliations:** 1 Department of Anesthesiology and Pain Medicine, Kangnam Sacred Heart Hospital, Hallym University College of Medicine, Seoul, Republic of Korea; 2 Department of Biostatistics, Yonsei University College of Medicine, Seoul, Republic of Korea

**Keywords:** Predictive markers, Diagnostic markers

## Abstract

Recent research has revealed that hemodynamic changes caused by lung recruitment maneuvers (LRM) with continuous positive airway pressure can be used to identify fluid responders. We investigated the usefulness of stepwise LRM with increasing positive end-expiratory pressure and constant driving pressure for predicting fluid responsiveness in patients under lung protective ventilation (LPV). Forty-one patients under LPV were enrolled when PPV values were in a priori considered gray zone (4% to 17%). The FloTrac-Vigileo device measured stroke volume variation (SVV) and stroke volume (SV), while the patient monitor measured pulse pressure variation (PPV) before and at the end of stepwise LRM and before and 5 min after fluid challenge (6 ml/kg). Fluid responsiveness was defined as a ≥ 15% increase in the SV or SV index. Seventeen were fluid responders. The areas under the curve for the augmented values of PPV and SVV, as well as the decrease in SV by stepwise LRM to identify fluid responders, were 0.76 (95% confidence interval, 0.61–0.88), 0.78 (0.62–0.89), and 0.69 (0.53–0.82), respectively. The optimal cut-offs for the augmented values of PPV and SVV were > 18% and > 13%, respectively. Stepwise LRM -generated augmented PPV and SVV predicted fluid responsiveness under LPV.

## Introduction

In patients undergoing general anesthesia and mechanical ventilation, dynamic preload indices such as stroke volume variation (SVV) and pulse pressure variation (PPV) are widely accepted as most accurate indicators of fluid responsiveness^1–3^. Many studies revealed that goal-directed fluid therapy based on these indices can help lower the risk of surgical complications by identifying the patients who can benefit from fluid administration^4,5^. However, the accuracy of PPV and SVV is reliable only when physiologic limitations on their use are followed^6,7^, and might be influenced by a variety of clinical and technical factors^8–10^. These indices are calculated from fluctuations in stroke volume (SV) produced by periodic intrathoracic pressure changes. As a result, the accuracy of PPV and SVV may be impaired by decreased pressure transfer from the lungs to the intrathoracic vascular compartment, which is associated with use of low tidal volume (VT less than 8 ml/kg)^11^, low pulmonary compliance^8,12^, and low driving pressure^13^. Lung-protective ventilation (LPV) with low VT has been shown to improve postoperative outcomes in surgical patients^14,15^, and is now recommended as the standard ventilation strategy in the operating room (OR)^16^. As a result, the applicability of these dynamic variables in routine clinical practice may be limited.

As part of LPV, intra-operative lung recruitment maneuvers (LRMs) are used to reopen collapsed lungs and enhance arterial oxygenation by applying high peak inspiratory pressure (PIP)^17,18^. Increased PIP during LRM reduces venous return and increases pulmonary vascular resistance, both of which cause a decrease in SV^19^. Studies have demonstrated that patients who are preload-dependent are more susceptible to these effects of LRM^20,21^. As a result, LRM can reveal hidden preload dependencies, aiding in the differentiation of fluid responders from non-responders. Recent studies have shown that the degree of hemodynamic alterations caused by LRMs with continuous positive-airway pressure (CPAP) or "manual bag squeezing" can be used to identify fluid responders in patients under LPV. However, LRMs with CPAP can cause hemodynamic instability and over-stretch healthy alveoli, leading to ventilator-induced secondary lung injury^22,23^. In comparison to CPAP-based techniques, stepwise LRM with increasing positive end-expiratory pressure (PEEP) and constant driving pressure is less likely to generate lung injury because it entails a gradual approach to maximum PIP^24,25^. Recently, new software has been integrated into anesthesia machines to perform stepwise LRM automatically, making it easier to use in clinical settings^26^.

The aims of the current study were to evaluate the ability of a new functional test using automated stepwise LRM for distinguishing fluid responders from non-responders in patients undergoing LPV in the OR. We hypothesized that the augmentation of PPV and SVV values and/or decrease in SV generated by increased PIP during stepwise LRM would be more prominent in hypovolemic patients.

## Methods

### Ethical approval

On September 10, 2019, the Institutional Review Board of Hallym University Kangnam Sacred Heart Hospital in Seoul, Korea (Chairperson Prof. Ik Yang) granted ethical approval for this prospective, observational study (approval number: 2019-07-004). Prior to patient enrolment, the trial was registered at ClinicalTrials.gov (reference number NCT04118244, date of registration 2 October 2019, principal investigator J-HJ). All the study procedures adhered to the 2013 Declaration of Helsinki. All study participants provided their informed consent.

### Patient population

Adult patients scheduled for elective laparotomy were enrolled between October 2019 and October 2021. To reduce the risk of complications from unnecessary LRM and fluid challenge (FC), patients were only enrolled if their PPV value was within a previously defined gray zone (between 4 and 17%)^27^. Patients with chronic obstructive pulmonary disease, preoperative arrhythmia, severe bradycardia, preoperative left ventricular (LV) ejection fraction less than 50%, right ventricular (RV) dysfunction, moderate to severe valvular heart disease, moderate to severe liver or renal disease, or body mass index less than 15 kg m^–2^ or greater than 30 kg m^–2^ were excluded.

### Anesthesia preparation

After admission to the OR, standard monitoring, including pulse oximetry, three-lead ECG, and non-invasive blood pressure, was applied. Anesthesia induction was performed with a bolus injection of propofol (1.5–2.0 mg/kg) and continuous infusion of remifentanil (0.1 μg/kg/min). Muscle relaxation was facilitated with rocuronium (0.7 mg/kg). Anesthesia was maintained with desflurane and remifentanil to target the state and response entropy values between 40 and 60. Patients were ventilated using the Datex-Ohmeda anesthetic ventilator (Avance CS^2^ Anesthesia Machine; GE Healthcare, Helsinki, Finland) in volume-controlled mode as follows: VT of 6 ml per predicted body weight (PBW)^28^, PEEP of 5 cmH_2_O, inspiratory to expiratory time (I:E) ratio of 1:2 without inspiratory pause, inspired oxygen fraction of 0.5, and respiratory rate (RR) set to target the end-tidal carbon dioxide tension of 35‒40 mmHg.

### Hemodynamic monitoring

An arterial catheter was cannulated into the radial artery and connected to a FloTrac transducer (Edwards Lifesciences, Irvine, CA, USA). After zeroing the pressure transducer to ambient atmospheric pressure at the mid-thoracic level, a fast-flush test was conducted to confirm that the arterial pressure wave system was not damped. Both the Vigileo monitor (Edwards Lifesciences, Irvine, CA, USA) and the patient monitor (CARESCAPE Monitor B850; GE Healthcare) received the arterial pressure signal at the same time. The patient monitor showed a real-time PPV value, which was calculated automatically every 5 s using the algorithms described previously^29^.

$${\text{PPV }}\left( \% \right) \, = \, \left[ {\left( {{\text{PP}}_{{{\text{max}}}} {-}{\text{ PP}}_{{{\text{min}}}} } \right)/{\text{PP}}_{{{\text{mean}}}} } \right] \, \times { 1}00,$$

where PP_max_ and PP_min_ represent the maximum and minimum arterial pulse pressure (PP), and PP_mean_ is the mean arterial PP.

The Vigileo-FloTrac device (Version 4.0, Edwards Lifesciences, Irvine, CA, USA) showed the values of SV, stroke volume index (SVI), and SVV, which was calculated by continuously analyzing the arterial waveform without every 20 s external calibration^30^: The SVV was calculated as follows:

$${\text{SVV }}\left( \% \right) \, = \, [({\text{SV}}_{{{\text{max}}}} {-}{\text{SV}}_{{{\text{min}}}} )/{\text{SV}}_{{{\text{mean}}}} ] \, \times { 1}00,$$

where SV_min_ and SV_max_ represent the minimum and maximum SV values and SV_mean_ is the mean SV.

Intra-operative hemodynamic data [heart rate, mean arterial pressure (MAP), SV, SVI, PPV, and SVV) and ventilatory data [VT, RR, PIP, PEEP level, and compliance of the respiratory system (Crs) were collected from the patient monitor, anesthesia machine, and Vigileo monitor. The data were recorded in a time-synchronized manner on a computer using data acquisition software (Vital Recorder, VitalDB; https://vitaldb.net/vital-recorder/)^31^, and was reconstructed with 5-s intervals into a dedicated EXCEL (Microsoft, Redwood, Mississippi, USA) spreadsheet for further analysis.

### Study protocol

**Figure 1:**
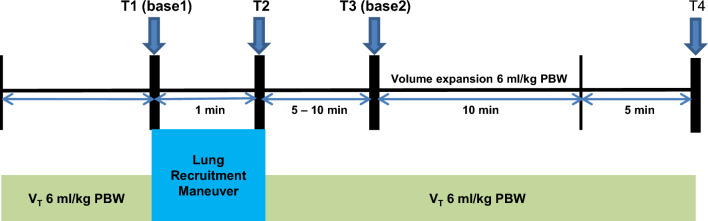
Study protocol. Four sets of hemodynamic and ventilatory variables were measured immediately before LRM (base 1, T1), at the end of LRM (LRM, T2), before VE (base 2, T3), and 5 min after VE (T4). *VT* tidal volume, *PBW* predicted body weight.

**Figure 2:**
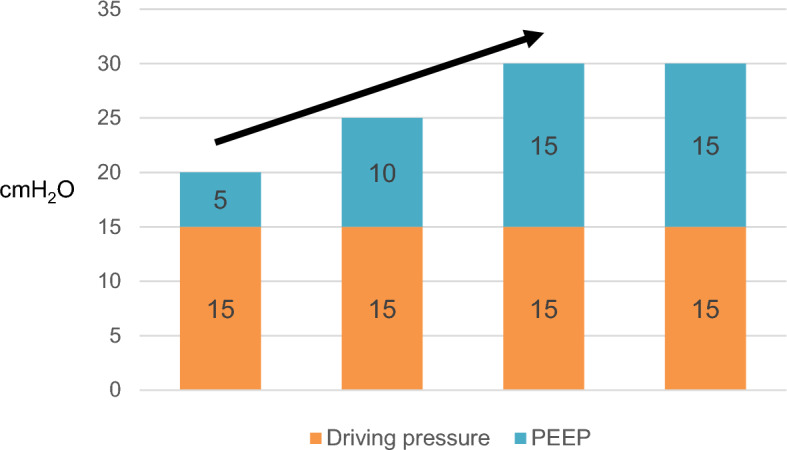
Representative method of the stepwise lung recruitment maneuver (LRM) using incremental positive end expiratory pressure (PEEP) at a constant driving pressure.

The study protocol (Fig. 1) was initiated after a 5-min hemodynamic stabilization period following peritoneum closure to minimize the effect of abrupt changes in sympathetic tone that may occur^32,33^. When a patient met the inclusion criteria, a stepwise LRM procedure was carried out by anesthesia ventilator using a predetermined time period and inflation pressure, which was performed based on previous study^34^. In brief, LRM was performed under pressure-controlled ventilation (PCV) with constant driving pressure (PIP minus PEEP) of 15 cmH_2_O, I:E ratio of 1:1, and RR of 12 per min. PEEP was increased in increments of 5 cmH_2_O every 3 breaths, from 5 to 15 cmH_2_O. The final recruitment pressure of 30 cmH_2_O was maintained for 6 breaths. The LRM lasted for 60 s (Fig. 2). After LRM, ventilatory settings were automatically returned to their initial settings. FC was performed immediately after hemodynamic variables returned to baseline (variation 10%) by infusing a balanced crystalloid solution in a volume of 6 ml per PBW for 10 min. To assess the hemodynamic effect of LRM and FC, the time synchronized data were extracted from the data set collected during surgery at four time points: immediately before LRM (base 1, T1), for 20 s after the end of LRM, (T2), before VE (base 2, T3), and at 5 min after VE (T4) (Fig. 1). At T2, the most decreased MAP and SV values, as well as the most augmented PPV and SVV values, were recorded; at all other times, the average of three consecutive measurements was recorded. LRM-induced SV change (between T1 and T2) (ΔSV_LRM_) was calculated as follows:

$$\Delta {\text{SV}}_{{{\text{LRM}}}} = \, \left[ {\left( {{\text{SV at T2 }}{-}{\text{ SV at T1}}} \right)/{\text{SV at T1}}} \right] \, \times {1}00.$$

According to the percentage changes in SV and SVIs after FC (between T3 and T4), patients were considered as fluid responders to VE if SV or SVI increased by ≥ 15% and as non-responders if they increased by < 15% of the baseline values^35^.

Thirty-eight patients were needed to detect the difference between the area under the receiver operating characteristic (ROC) curve (AUC) under the null hypothesis of 0.5 and the alternative hypothesis of 0.75 with 80% power, a significance threshold of 5% and two-sided testing, assuming an equal number of fluid responders and non-responders^36^. To account for a potential 20% dropout rate, 48 participants were enrolled in this trial.

### Statistical analysis

The distribution of continuous data was tested using the Shapiro–Wilk test. Data comparisons between responders and non-responders were performed using an independent *t*-test, Mann–Whitney *U*-test, or χ^2^ tests, depending on the data characteristics. Data were expressed as mean ± SD, median [interquartile range (IQR)], or number of patients (%). The effects of LRM and VE on the hemodynamic parameters were assessed using a linear mixed model. The fixed effects were the time of assessment, treatment (responders and non-responders), and treatment-by-time interaction. Multiple comparisons were controlled using a Bonferroni-adjusted P-value, which is the raw *P*-value multiplied by the number of outcomes being tested. The relationship between the hemodynamic variables before VE and percentage changes in SV by VE were evaluated using Spearman’s rank correlation analysis. A random effects model was used to evaluate the intra-class correlation between the SV measurements at base 1 (T1) and base 2 (T3)^37^. To test the abilities of hemodynamic variables before FC in predicting fluid responsiveness, the AUCs of ROC curves were calculated using the Hanley & McNeil method. An AUC greater than 0.75 was considered to indicate good diagnostic value^38^. An optimal cut-off value was determined for each variable that could maximize the Youden index (sensitivity + specificity–1).

MedCalc (version 20.111; MedCalc Software, Ostend, Belgium) and SAS (version 9.4; SAS Institute Inc., Cary, NC, USA) were used to perform statistical analyzes. In all analyzes, statistical significance was set at *P* < 0.05.

## Results

**Figure 3:**
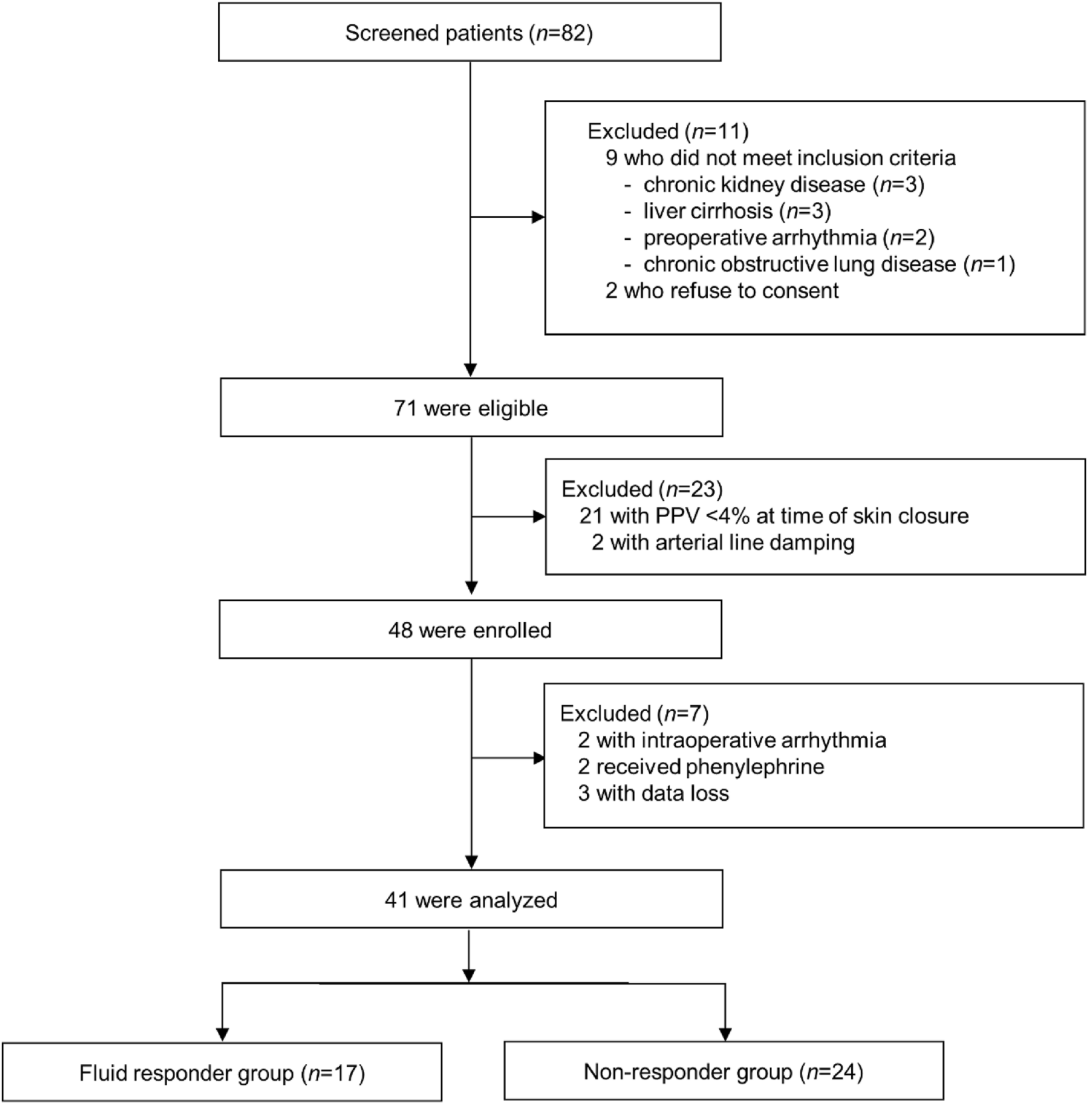
Flow diagram of our study.

Of the 82 patients assessed for eligibility between October 2019 and October 2021, 48 met the inclusion criteria and were enrolled. Seven patients were subsequently excluded: two developed unexpected arrhythmias after baseline measurement, two suffered hypotension after LRM and required vasopressor support, and three reported data loss. In total, 41 patients were included in the final analysis, and 17 were classified as responders (41.5%) and 24 as non-responders (58.5%) (Fig. 3). The baseline characteristics of the patients are presented in Table 1. The intra-class correlation coefficient between SV measurements at the two baseline steps (T1 and T3) was 0.99 (95% CI 0.99 to 1.0).

**Table 1:** Patient characteristics.

	Overall (*n* = 41)	Responder (*n* = 17)	Non-responder (*n* = 24)
Age (years), mean (SD)	59.7 (8.8)	62.9 (9.2)	57.4 (7.9)
Male, n (%)	26 (63.4%)	9 (52.9%)	17 (70.8%)
PBW (kg), mean (SD)	58.6 (10.0)	55.0 (11.4)	61.2 (8.3)
BMI (kg·m^–2^), mean (SD)	22.9 (2.8)	22.7 (3.0)	23.1 (2.7)
Hypertension, n (%)	15 (36.6%)	6 (35.3)	9 (37.5)
Diabetes mellitus, n (%)	10 (24.4%)	5 (29.4)	5 (20.8)
Medication, n (%)			
Beta blocker	1 (2.4%)	0 (0%)	1 (4.2%)
Calcium channel blocker	9 (22%)	3 (17.6%)	6 (25%)
Diuretics	4 (9.8%)	2 (11.8%)	2 (8.3%)
ARB	13 (31.7%)	5 (29.4%)	8 (33.3%)
Surgery type, n (%)			
Colectomy	9 (22%)	4 (23.5%)	5 (20.8%)
Hepatectomy	6 (14.6%)	3 (17.6%)	3 (12.5%)
Pancreatoduodectomy	9 (22%)	4 (23.5%)	5 (20.8%)
Distal pancreatectomy	2 (4.9%)	2 (11.8%)	0 (0%)
Gastrectomy	13 (31.7%)	3 (17.6%)	10 (41.7%)
Ileostomy	2 (4.9%)	1 (5.9%)	1 (4.2%)

*PBW* predicted body weight, *BMI* body mass index, *ARB* Angiotensin receptor blocker, *SD* standard deviation.

### Effects of LRM and FC on hemodynamic and respiratory variables

**Figure 4:**
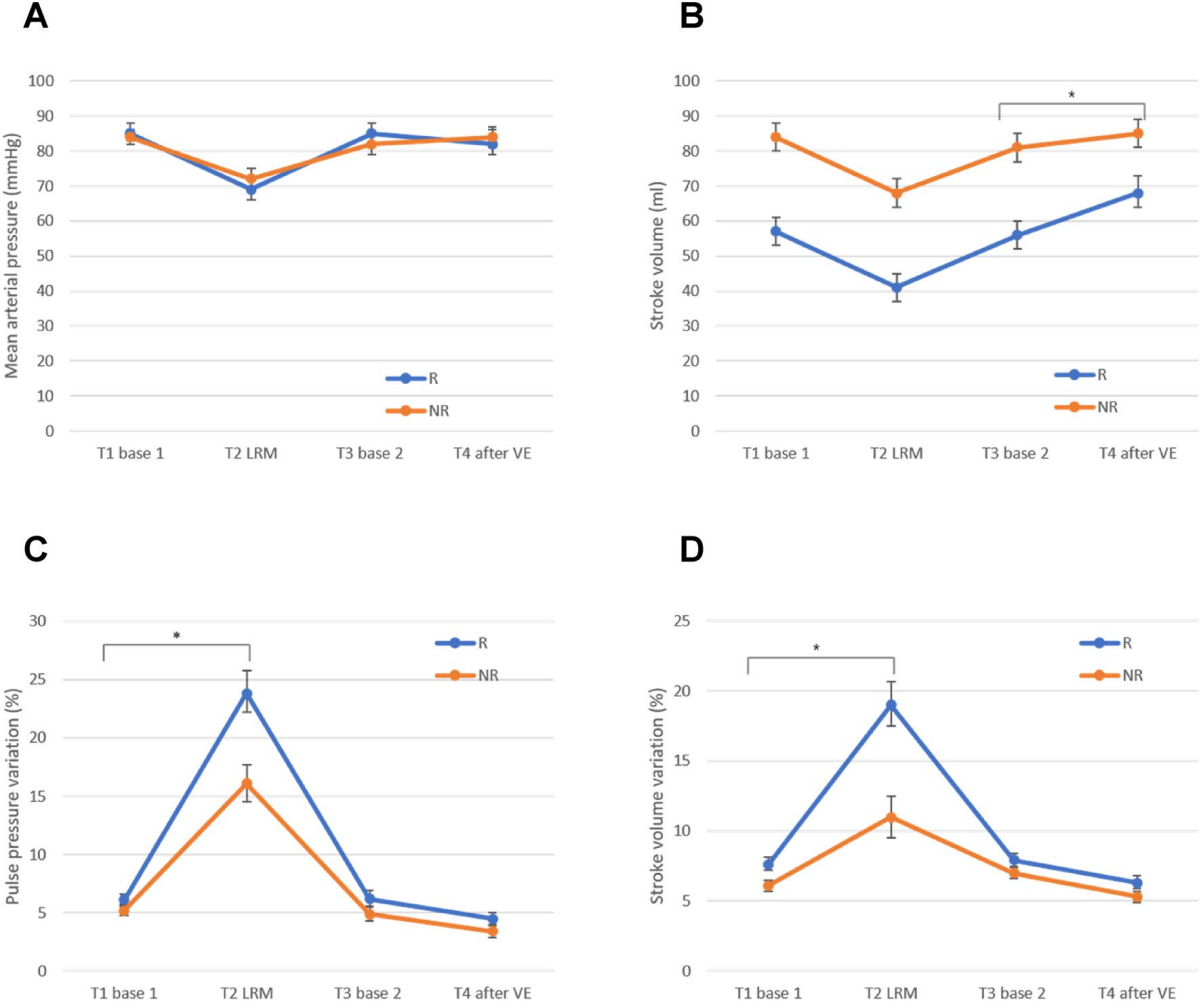
Change in mean arterial pressure (**A**), stroke volume (**B**), pulse pressure variation (**C**), and stroke volume variation (**D**) at four times points, from T1 to T4. T1, immediately before lung recruitment maneuver (LRM); T2, at the end of LRM; T3, 5 min after LRM; T4, 5 min after fluid challenge (FC). *P < 0.05 for group and time interaction.

LRM induced a significant decrease in MAP and SV and a significant augmentation of PPV and SVV in both responders and non-responders (Table 2). The LRM-induced decrease in MAP and SV did not differ between responders and non-responders, whereas LRMs induced significantly larger PPV and SVV augmentation in responders than in non-responders (Fig. 4). LRM resulted in significant increases in PIP and Crs in both responders and non-responders (Table 2), with no difference between the two groups (*P* = 0.20 and *P* > 0.99, respectively.

**Table 2:** Hemodynamic and ventilatory data at each time point of the study protocol in responders (*n* = 17) and non-responders (*n* = 24).

	T1, base 1	T2, LRM	P1^†^	T3, base 2	T4, after FC	P2^‡^
HR (bpm)						
Responders	80 (3)	77 (3)	> 0.99	80 (3)	74 (3)	< 0.001
Non-responders	75 (3)	73 (3)	0.24	74 (3)	72 (3)	0.03
MAP (mmHg)						
Responders	85 (3)	69 (3)	< 0.001	85 (3)	82 (4)	> 0.99
Non-responders	84 (2)	72 (3)	< 0.001	82 (3)	84 (3)	> 0.99
SV (ml m^–2^)						
Responders	57 (4)*	41 (4)*	< 0.001	56 (4)*	68 (5)	< 0.001
Non-responders	84 (4)*	68 (4)*	< 0.001	81 (4)*	85 (4)	0.01
PPV (%)						
Responders	6.1 (0.5)	23.8 (2.0)*	< 0.001	6.2 (0.7)	4.5 (0.5)	0.01
Non-responders	5.2 (0.4)	16.1 (1.6)*	< 0.001	4.9 (0.6)	3.4 (0.5)	0.01
SVV (%)						
Responders	7.6 (0.5)	19 (1.7)*	< 0.001	7.9 (0.5)	6.3 (0.5)	0.01
PIP (cm H_2_O)						
Responders	14 (0.3)	30 (0)	< 0.001	14 (0.2)	14 (0.2)	0.27
Non-responders	15 (0.2)	30 (0)	< 0.001	14 (0.2)	15 (0.2)	0.04
Crs (ml/cmH_2_O)						
Responders	36 (1.4)	46 (2)	< 0.001	37 (1.5)	35 (1.5)	0.16
Non-responders	36 (1.2)	47 (1.7)	< 0.001	39 (1.3)	37 (1.3)	0.24

Data are presented as the estimated mean (SE).

*Crs* compliance of the respiratory system, *HR* heart rate, *LRM* lung recruitment maneuver, *MAP* mean arterial pressure, *PIP* peak inspiratory pressure, *PPV* pulse pressure variation, *SV* stroke volume, *SVV* stroke volume variation, *FC* fluid challenge.

*Bonferroni-corrected *P* < 0.05 comparison between responders and non-responders at each time point.

^†^*P1*- adjusted P values (using Bonferroni correction) for intragroup comparisons of values before (T1) and after lung recruitment (T2).

^‡^P2- adjusted P values (using Bonferroni correction) for intragroup comparisons of values before (T3) and after fluid challenge (T4).

FC induced a significant increase in SV and a significant decrease in PPV and SVV in both responders and non-responders (Table 2). VE-induced decrease in PPV and SVV did not differ between responders and non-responders (Fig. 4).

### Relationships between LRM-induced changes in hemodynamic variables and FC-induced percentage changes in SV

PPV and SVV at T2 (end of LRM) showed moderate correlation with FC-induced percentage changes in SV [*r* = 0.45 (95% CI 0.17 to 0.67), *P* = 0.003; and *r* = 0.54 (95% CI 0.28 to 0.73), *P* < 0.001, respectively], whereas SVV at T1 (base 1) and ΔSV_LRM_ showed low correlation [*r* = 0.31 (95% CI 0.01 to 0.57), *P* = 0.046; and *r* = 0.38 (95% CI 0.09 to 0.62), *P* = 0.013; respectively]. PPV at T1 did not correlate with the FC-induced percentage changes in SV [*r* = 0.17 (95% CI 0.14 to 0.46), *P* = 0.27].

### Ability of hemodynamic changes during LRM to predict fluid responsiveness

**Figure 5:**
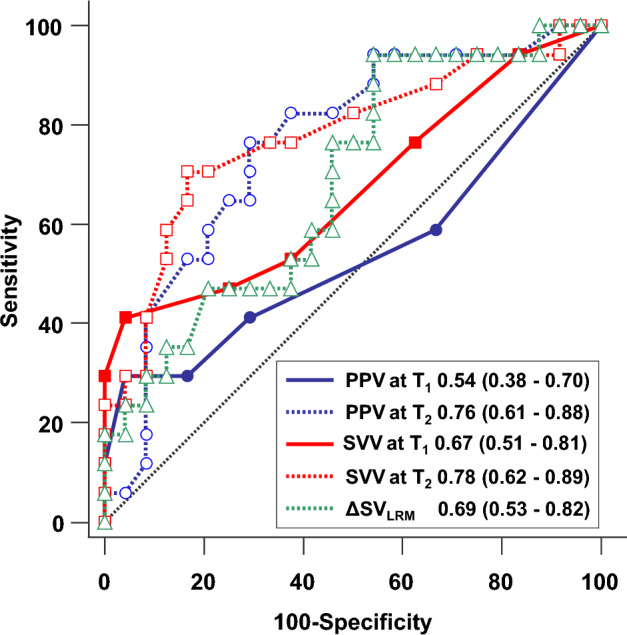
Receiver-operating characteristic curves comparing the abilities of PPV at T1, SVV at T1, PPV at T2, SVV at T3, and ΔSV_LRM_ to predict fluid responsiveness under lung-protective ventilation. PPV at T1, baseline value of pulse pressure variation; PPV at T2, augmented value of pulse pressure variation induced by lung recruitment maneuver; SVV at T1, baseline value of stroke volume variation; SVV at T2, augmented value of stroke volume variation induced by lung recruitment maneuver; ΔSV_LRM_, LRM-induced changes in stroke volume.

Figure 5 shows the ROC analysis for identifying fluid responders. The AUCs for PPV and SVV at T2 (end of LRM) in distinguishing fluid responders from non-responders were 0.76 (95% CI 0.61 to 0.88, P = 0.0006) and 0.78 (95% CI 0.62 to 0.89, P = 0.0003), respectively. In contrast, neither PPV nor SVV at T1 (base 1) could distinguish fluid responders from non-responders, with AUCs of 0.67 (95% CI 0.51–0.81, P = 0.06) and 0.54 (95% CI 0.38 to 0.70, P = 0.067), respectively. The optimal cut-off values for the PPV and SVV at T2 (end of LRM) were 18% [sensitivity of 76% (95% CI 50 to 93), with specificity of 71% (95% CI 49 to 87)] and 13% [sensitivity of 71% (95% CI 44 to 90) and specificity of 83% (95% CI 63 to 95)], respectively. The AUC for ΔSV_LRM_ to distinguish fluid responders from non-responders was 0.69 (95% CI 0.53 to 0.82, *P* = 0.024). The optimal cut-off value for ΔSV_LRM_ was 16% with a sensitivity of 94% (95% CI 71 to 100) and specificity of 46% (95% CI 26 to 67).

## Discussion

In the present study on patients within the gray zone (PPV values between 4 and 17%) during LPV using low VT, the augmented values of PPV and SVV generated by automated stepwise LRM could predict fluid responsiveness, whereas baseline PPV and SVV could not distinguish fluid responders from non-responders. The optimal cut-offs for the augmented PPV and SVV were > 18% and > 13%, respectively. A 16% decrease in SV during LRM could also distinguish fluid responders from non-responders; however, it showed poor diagnostic value.

### Improving the predictability of PPV and SVV through stepwise LRM

Under LPV using low VT (less than 8 kg^–1^ of PBW), the variations in LV SV induced by periodic changes in PIP are reduced, which affects the accuracy of dynamic preload indices in predicting fluid responsiveness^11,39^. A previous experimental animal study demonstrated that the value of SVV augmented with the increase in PIP, and this association was more prominent in a decreased preload state^40^. Thus, we hypothesized that increased PIP during stepwise LRM would augment the values of dynamic preload indices, which would contribute to improving their ability to identify fluid responders. A similar strategy had previously been successfully explored in patients under open-chest settings, which influences the dynamic preload index similarly to low VT by minimizing the effect of periodic variations in PIP on LV SV. In this study by Min et al.^41^, augmented PPV values by LRM with CPAP at 30 cmH_2_O for 10 s had a better diagnostic value in predicting fluid responsiveness than standard PPVs, with an AUC of 0.88 (95% CI 0.75–0.95). This improvement in the ability to identify a fluid responder through augmentation of PPV values was also proven in our study.

### Optimal cutoffs of augmented PPV and SVV induced by stepwise LRM

Meanwhile, despite applying the same target recruitment pressure of 30 cmH_2_O in our study, the augmented PPV induced by stepwise LRM was lower than that induced by CPAP in the study by Min et al. in terms of optimal thresholds (18% vs. 55%)^41^. The main cause of this discrepancy may be the application of a constant airway driving pressure of 15 cmH_2_O while increasing PEEP in our study. Airway drive pressure is a well-known factor that has a considerable effect on the value and reliability of dynamic preload indices^13^. It is unknown, however, if PEEP influences the values and reliability of dynamic preload indices. Previous studies have reported that PPV was augmented with an increase in PEEP because of reduced SV^42,43^. However, in these studies, fixed VT ventilation was used, which caused the PEEP and driving pressure to change simultaneously^43^; thus, the effect of PEEP on PPV could not be confirmed. A recent experimental study using beagle dogs investigated the effect of PEEP on SVV under fixed airway driving pressure but not under fixed VT. In their experiment, PEEP had no effect on SVV value, which clarified that the SVV value was augmented by an increase in airway driving pressure rather than by an increase in PEEP^44^.

### Low diagnostic value of decrease in SV during stepwise LRM

Increased PIP during LRM reduces RV-SV by reducing venous return and increasing pulmonary vascular resistance^19^. Biais et al. identified a strong correlation between the magnitude of SV decrease induced by LRM using CPAP of 30 cmH_2_O for 30 s and that induced by VE in patients receiving LPV using low VT. A 30% decrease in SV during LRM accurately predicted a positive response to a 250 mL crystalloid VE with an AUC of 0.96 (95% CI 0.81–0.99)^14^. However, in our study, the stepwise LRM-induced changes in SV were unable to accurately identify the fluid responders. This disparity could be explained by the fact that we only studied patients in the gray zone, and our patients had higher baseline preload reserves than previous research, making it more difficult to detect fluid responders. Another explanation for this disparity is that the hemodynamic effects of LRM depend on the recruitment method. A recent study in preload-independent patients during anesthesia reported that stepwise LRM resulted in less profound changes in SV than through CPAP^19^.

### Fluid challenge

FC is the gold standard for evaluating fluid responsiveness However, there is significant variation in FC methods for the amount of fluid, the rate of administration, the type of fluid used, and when to assess the maximal effect of FC between trials, which may result in different changes in SV even in the same setting, and thus different proportions of patients considered responsive to an FC^45–48^.

If insufficient fluid is given during FC, no substantial hemodynamic changes will occur, making it difficult to identify the fluid responder^45,47,48^. We performed FC for 10 min using an infusion of balanced crystalloid solution (6 ml/ kg), as described in prior research in the intensive care unit and perioperative setting^49,50^. Recently, Aya et al. applied pharmacodynamic methodology in postoperative patients and showed that the minimal dose required for effective FC is 4 ml/kg^45^. A recent meta-analysis of variables influencing fluid responsiveness prediction also found that predictors performed better when FC exceeded 4 ml/kg^9^, implying that the dose and rate of FC utilized in our investigation were adequate to assess predictor performance.

Because there was limited literature evidence on the maximal effect of FC on SV at the time of planning this study, the effect of FC was evaluated 5 min after the end of fluid infusion, as in previous investigations^49,51,52^. However, a recent pharmacodynamic analysis of the hemodynamic effects of a 250 ml crystalloid FC revealed that the maximum change in cardiac output occurs at least 1 min after the fluid infusion ends, and that the effect on cardiac output is temporary, dissipating in 10 minutes^46^. As a result, the evaluation time point we chose may underestimate the FC's maximal effect, and responders may have been misclassified as non-responders, lowering the predictors' predictive value.

### Strength of study

The automated stepwise LRM utilized in this study has several advantages as a functional test for predicting fluid responsiveness. Stepwise LRM, unlike CPAP, is administered under PCV, allowing the augmented values of SVV and PPV to be automatically calculated and shown using the hemodynamic monitor's pre-existing algorithms^41,53^. Another benefit is that LRM was provided to all patients at a consistent pressure for a constant period utilizing the anesthetic machine's automated features, allowing for a more quantitative measurement of hemodynamic changes produced by increased PIP during LRM than earlier studies with manual LRM^26^.

### Limitation of study

Despite the advantages described above, our functional tests have some limitations. First, changes in SV induced by LRM and VE measured using were estimated using Vigileo/ FloTrac system. Arterial compliance affects this system's SV measurement accuracy^30^. Arterial compliance has an impact on this system's SV measurement accuracy. Changes in arterial compliance during LRM may have an impact on the precision of measured SV, which could be a significant limitation. Second, we assessed the ability of a functional test using stepwise LRM during the steady-state period after peritoneal closure. Thus, it is unclear whether our findings can be generalized to laparotomy with an open peritoneum. Our results require validation in other clinical scenarios. Third, despite their apparent benefits, LRMs are not without risk. The routine use of LRMs during the intra-operative phase is discouraged by current guidelines because they potentially harm the hemodynamic and do not improve clinical outcomes^16^. Furthermore, because PPV values below or above the gray zone are known to be highly accurate in predicting fluid responsiveness in mechanically ventilated patients without spontaneous breathing or arrhythmias^6,7^, functional tests to assess fluid responsiveness are unnecessary if a patient's PPV is not in the gray zone. Our functional test should not be utilized as a stand-alone tool for fluid responsiveness assessment; rather, it should be used in conjunction with other preload-dependent data (such as hemodynamic parameters, dynamic preload indices, and SV changes after FC). Forth, the gray zone applied in this study was the suggested value in an intensive care unit setting because, to date, no study has been conducted on the gray zone of PPV in a large population in a surgical setting under low VT ventilation^27^. If a precise range of the gray zone of PPV in a large surgical population undergoing low VT ventilation is known through additional studies, our functional test can be accurately applied only when there is an additional benefit.

## Conclusion

The augmented values of PPV and SVV generated by automated stepwise LRM can predict fluid responsiveness in patients within the gray zone under LPV. Therefore, if used carefully in clinical practice, taking the idea of the gray zone into account, this functional test may help optimize perioperative fluids in patients under LPV, contributing to improved clinical outcomes. Additional research involving a larger sample size and more examiners is required to validate our findings.

## Data Availability

Due to ethical restrictions, the datasets used and/or analyzed during the current study are available from the corresponding author on reasonable request.
